# A community-based ambulance model: lessons for emergency medical services and everyday health systems resilience from South Africa

**DOI:** 10.1093/heapol/czae070

**Published:** 2024-08-02

**Authors:** Leanne Brady, Lucy Gilson, Asha George, Shaheem De Vries, Shakira Hartley

**Affiliations:** Health Policy and Systems Division, School of Public Health, University of Cape Town, Cape Town 7700, South Africa; Western Cape Department of Health and Wellness, Cape Town 8000, South Africa; Health Policy and Systems Division, School of Public Health, University of Cape Town, Cape Town 7700, South Africa; Department of Global Health and Development, London School of Hygiene and Tropical Medicine, London WC1E 7HT, United Kingdom; School of Public Health, University of the Western Cape, Cape Town 7460, South Africa; South African Medical Research Council, Cape Town 7505, South Africa; Western Cape Department of Health and Wellness, Cape Town 8000, South Africa; Western Cape Department of Health and Wellness, Cape Town 8000, South Africa

**Keywords:** Ambulance, community-based, everyday resilience, resilience capacities

## Abstract

The role of the emergency medical service (EMS) is changing globally as ambulance crews respond to a shifting burden of disease, as well as societal stressors such as violence and inequality. New ways of thinking about how to provide emergency care are required to shift EMS from a role primarily focused on clinical care and transporting patients to hospital. In this paper, we present the experience of the Philippi Project (PP), an innovative community-based model of care developed by front line ambulance crews in a low-income neighbourhood in Cape Town, South Africa. Our insights were developed through observational, interview and document review work, within an overall embedded research approach. Our analysis draws on the everyday health systems resilience (EHSR) framework, which sees resilience as an emergent process that may be stimulated through response to stress and shock. Responses take the form of absorptive, adaptive or transformative strategies and are underpinned by system capacities (cognitive, behavioural and contextual). We consider the PP as a potentially transformative resilience strategy, defined as a new way of working that offered the promise of long-term health system gains. We found that the PP’s initial development was supported by a range of system capacity attributes (such as the intentional development of relationships, a sense of collective purpose and creating spaces for constructive sense-making). However, the PP was hard to sustain over time because emergent ways of working were undermined both by other capacity attributes rooted in pre-existing organizational routines and two contextual shocks (Coronavirus and a violent incident). The paper adds a new empirical contribution to the still-small EHSR literature. In addition, the PP experience offers globally relevant lessons for developing community-based models of EMS care. It demonstrates that front line staff can develop creative solutions to their stressful daily realities, but only if space is created and protected.

Key messages

- Existing emergency medical service routines initially established to support a hospi-centric service may no longer be sufficient. Responding to complex social stressors and developing community-based models of care require new ways of thinking and doing.
- It is important to create and protect spaces for frontline staff to develop creative, collective solutions to the daily stressors (such as violence and inequality) they face.
- Although individuals can initiate responses to health system shocks and stressors, longer-term resilience strategies require collective engagement and action to be sustained.
- Everyday resilience capacities are embedded in people, teams and system routines and must be spread across all levels of the health system.

## Introduction

Across the world, the role of the emergency medical service (EMS) is changing (NHSS, 2015; Eaton *et al*., 2018). Historically focused on emergency care and patient transport to hospitals, paramedics have increasingly been expected to respond to a broader array of health and social care needs (Eaton *et al*., 2018). In some countries, there has been a shift towards a community-based service (EMS, 1996; NCSL, 2022) that is better integrated with the primary health care platform (Ball, 2005), including the development of a new EMS cadre, community paramedics (Kizer and Moulin, 2013; Rasku *et al*., 2019). The future of EMS has even been envisaged by some as a ‘safety net’ (EMS, 1996), playing a broader role in relation to societal injustices including gender and race inequalities (Tavares *et al*., 2022).

However, very little research worldwide has considered experience in adopting new EMS models of care. In low- and middle-income countries, research has largely focused on in-hospital emergency care (Obermeyer *et al*., 2015), while work on pre-hospital or out-of-hospital services has focused predominantly on protocols for improved clinical care (McCaul *et al*., 2016), access (Stein *et al*., 2016; Marsh and Rouhani, 2018), response times (Vanderschuren and McKune, 2015; Vincent-Lambert and Mottershaw, 2018) and hardware issues such as infrastructure gaps (Hsia *et al*., 2012). Additional research priorities on pre-hospital and emergency care in sub-Saharan Africa (Mould‐Millman *et al*., 2013) and South Africa (van Hoving *et al*., 2015) include evaluating home-grown local systems of care and quality improvement (Howard *et al*., 2020). However, no research to understand the evolving role of EMS in offering community-based care or what system capacities enable or constrain implementation of such care has been done.

In South Africa, ambulance services are offered by both the public and private sectors. Reflecting wider health system inequity (Ataguba and McIntyre, 2012), private ambulances cater largely to the small, insured population and primarily service private hospitals. In contrast, the public sector EMS has a constitutional mandate to ensure that no-one is denied access to emergency health care, irrespective of their race, class, nationality or ability to pay. Public sector EMSs are managed by provincial governments and operate under significant resource constraints. For example, South Africa currently has <50% of the ambulances it needs (Kahn, 2022). In the Western Cape specifically, there are only 130 operational ambulances (EMS, 2022) for an estimated population of 7.2 million people (StatsSA, 2022).

**Figure 1.**
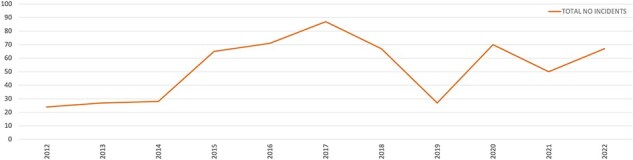
Total numbers of EMS staff assaulted per year (EMS, 2023)

As globally, the changing burden of disease in South Africa is demanding new ways of thinking about how to provide EMS (Achoki *et al*., 2022). In the Western Cape province, the 2019 Burden of Disease Report found that intentional injuries are the leading cause of years of life lost in men from low-income neighbourhoods across the Cape Town metro (Davies *et al*., 2019). This represents a significant disease burden, and the spatial distribution (Davies *et al*., 2019) reflects the societal inequality and fragmentation that remains a core legacy of apartheid even today. Societal violence resulting from such inequality also poses considerable safety risks for EMS staff. As demonstrated in Figure 1, the assaults on staff per year began to increase in 2014 and spiked to 87 in 2017 (EMS, 2023). Besides a decrease during the peak of the Coronavirus (COVID-19) pandemic, assaults on ambulance crews have remained a significant issue. Consequently, the provincial government has implemented various responses to address this situation. These included the ‘red zone policy’ that requires EMS crews to secure armed police escorts before entering neighbourhoods declared as particularly dangerous, as well as novel community-based models of care.

This paper reports the experience of one such model in the Western Cape, the Philippi Project (PP)—an initiative developed by frontline ambulance crews in response to their daily reality of social inequality and violence in a low-income urban neighbourhood in Cape Town. Considering the PP as a response to these everyday stressors, we first describe this collaborative initiative and its intentions, and then, using the everyday health systems resilience (EHSR) framework (outlined in the Methods section), we analyse the factors (understood as system capacities in the language of EHSR) that enabled and restrained it. Ultimately, the paper seeks to offer insights into the system-level capacity attributes needed to support innovative community-based models of EMS care.

## Methods

### Study setting

Philippi is one of the largest suburbs on the Cape Flats in Cape Town, South Africa. It is a mixture of farmlands and residential areas home to multiple communities (Narshai, 2019). All have been subject to a tumultuous history of land contestation. Over 2000 years before the first colonial encounter, the area was used for cattle grazing by the nomadic Khoekhoe (Adams *et al*., 2020). Then, after multiple waves of colonial invasions by the Dutch in 1656, the British in 1795 and then the German in 1877 (Battersby-Lennard and Haysom, 2012), its population was subject to ‘forced removals’ (Lindow, 2017) when the area was declared ‘White’ as a result of the apartheid Group Areas Act in the 1950s. During the 1990s, it became the site of violent protests against the allocation of housing for Black Africans leading to it being called an ‘Apartheid Battlefield’ (Narshai, 2019). Today, structural and spatial violence and inequality persist. It was designated an EMS ‘red zone’ in 2016. The most recent available census data (from 2011) found that 56% of its population of 191 015 lived in informal housing, with 44% in formal housing with services, and 52% of people lived below the poverty line (COCT, 2013).

### Conceptual framing

To support our analysis, we draw on the EHSR framework (Gilson *et al*., 2017; Barasa *et al.*, 2018). This framework recognizes that health systems face multiple everyday challenges even before the shocks of infectious disease or natural disasters impact on them. Using the language of the framework, the burdens and risks of societal violence represent, in the Western Cape, a form of chronic stress, an enduring reality to which the health system must respond to keep functioning. The framework calls responses to such stress, resilience strategies, and recognizes them as the collective actions of individuals and teams working in health systems, and, sometimes, across organizational boundaries. ‘Transformative’ strategies, specifically, can be understood as ‘new ways of working or new practices that offer the promise of significant and sustained renewal, reorganisation and development over the long term’ (Gilson *et al*., 2024, p. 11). As the PP sought to ensure that EMS could continue delivering emergency care in the face of the chronic stress of societal violence, we judged it to be a potentially transformative strategy. The framework also identifies three sets of systemic capacities that support (or in their absence, may constrain) collective responses to stress, as outlined in Table 1, and so influence the emergence of resilience over time.

**Table 1.** Summary of three sets of systemic capacities that support collective action, adapted from Weick *et al*. (2005), Lengnick-Hall *et al*. (2011) and Lengnick-Hall and Beck (2009)

Capacity	Description	Key attributes
Cognitive	The collective mental models and skills required to ‘notice, interpret, analyse and formulate a response to unfamiliar evolving situations’ as well as ‘contribute to the generation and selection of alternatives’ (Lengnick-Hall and Beck, 2009).	A ‘positive conceptual orientation’—a strong sense of purpose, authentic core values, genuine vision and deliberate use of language to frame conditions in ways that enable problem-solving and action (Lengnick-Hall and Beck, 2009) ‘Constructive sensemaking’—required to interpret situations, ‘generate and sustain shared meaning’ (Gilson *et al.*, 2024), which in turn provides a ‘springboard to action’ (Weick *et al*., 2005). ‘Purposeful framing’—use a vocabulary that implies capability, competence, consistent core values and a clear sense of direction to set the stage for sensemaking that enables problem-solving (Lengnick-Hall *et al*., 2011).
Behavioural	A toolbox of possible actions that can be collectively drawn upon in emerging situations—for example, to support an initial response to challenge that creates options rather than constrains future actions (Lengnick-Hall and Beck, 2009).	‘Learned resourcefulness, ingenuity and bricolage’ (the imaginative use of materials for previously unintended purposes)—enable groups to engage in the disciplined creativity needed to devise unconventional, yet robust, responses to unprecedented challenges (Lengnick-Hall *et al*., 2011)‘Act counterintuitively’—follow a dramatically different course of action from that which is the norm (Lengnick-Hall *et al*., 2011) ‘Useful, practical habits’—of investigation, collaboration, flexibility that become first response to unexpected events (Lengnick-Hall *et al*., 2011). ‘Behavioural preparedness’—making investments before they are needed so that the organization is able to benefit from situations as they emerge (Lengnick-Hall *et al*., 2011) ‘Dynamic tension’ between behaviours that foster creativity and unconventional actions and familiar and well-rehearsed routines that keep an organization grounded while providing a platform for inventiveness (Lengnick-Hall and Beck, 2009)
Contextual	The setting in which cognitive and behavioural capacities are brought to life. It is ‘the network of interactions and resources that provide the backdrop for an organization’s response to disruptive conditions’ (Lengnick-Hall and Beck, 2009) that are enabled through a foundation of interpersonal relationships.	‘Deep social capital’—developed through respectful social interactions within the organization—e.g. to share tacit knowledge—working across organizational boundaries and developing support networks. This is a key factor in enabling constructive sensemaking (Lengnick-Hall and Beck, 2009). ‘Broad resource networks’—relationships with others who could share key resources; assisting in providing continuous slack in the organization and extending the range of feasible actions (Lengnick-Hall and Beck, 2009) ‘Diffuse power and accountability’—to support self-organization, dispersed influence (Lengnick-Hall and Beck, 2009; Lengnick-Hall *et al*., 2011) ‘Psychological safety’—nurture an organizational context conducive to taking risks with freedom for experimentation (Lengnick-Hall *et al*., 2011).

In summary, the paper starts from the judgement that the PP is a potentially transformative EMS resilience strategy, a response to the everyday stressors associated with societal violence. It then seeks specifically to understand whether and how the systemic resilience capacities influenced this example of collective action.

### Embedded researcher role

The lead author (L.B.) is a medical doctor with 10 years of experience working within the Western Cape health system, as well as wider experience of contributing to community responses during the COVID-19 pandemic (Van Ryneveld *et al*., 2022). In 2019, she was appointed as an embedded researcher in EMS, the government ambulance service, and the research reported here was considered part of this role. Embedded research (Olivier *et al*., 2017) approaches require researchers to be located ‘within’ the health system and to do research ‘with’ and not ‘on’ health system actors (Olivier *et al*., 2017). They entail long-term actively facilitated processes of action and reflection (Lehmann and Gilson, 2014), supporting knowledge production and action at the interface of research and practice. They are increasingly acknowledged as offering support for health system strengthening (Vindrola-Padros *et al*., 2017; Marten *et al*., 2021).

L.B.’s role as part of the EMS leadership team, also engaging at district level, allowed insight into EMS debates and experience. The importance of better understanding the PP to inform EMS future planning became clear through this role. In addition, L.B.’s role as an embedded researcher enabled trusting relationships to be built with EMS colleagues, while her position as a medical doctor in an organization infused with clinical hierarchies afforded her a certain degree of unearned professional power. Together these two roles meant she was largely seen as a welcomed partial ‘insider’ (Yanow *et al*., 2009). They also enabled understanding of the phenomenon of interest and supported interpretative power in understanding the life-worlds of EMS staff involved in the PP (Yanow *et al*., 2009). However, it was necessary to constantly interrogate this position of familiarity, and to test and clarify her assumptions about how the PP crews experienced the daily realities of the system.

### Data collection

Drawing on methods from organizational ethnography (Yanow *et al*., 2009), data collection involved a series of in-depth interviews supplemented by observations, a research diary, a series of sense-making discussions with academic research team members (L.G. and A.G.) and wider reflection on insights gained as the embedded researcher in EMS, as summarized in Table 2.

**Table 2.** Data collection summary table

Data collection	Quantity	Primary focus (why it was done)	Details (what was done)
In-depth semistructured interviews	10	(1) To capture diverse narratives of the experience across a range of EMS actors to understand different perspectives. (2) To identify and explore the influence of the capacities over collective action.	Purposively sampled across EMS managerial ranks, and frontliners from across the system including ambulance crews, and safety desk colleagues from the communications centre.
Observations		To support a deeper exploration of the capacities and supplement the narratives from interview data.	Observations were split across day shift and night shift.
Ride-a-longs	Four shifts (3 days/1 night) 14 observational hours		
Safety desk	Four shifts (2 days/2 nights) 10 observational hours		
Research diary	1	To document observations, wider reflections and assist with deeper understanding of positionality.	Reflections captured throughout the research process. Specifically, detailed notes of the observations were made.
Research team discussions (University colleagues, L.G. and A.G.)	12	Sense-making sessions to draw in broader health systems experiences and literature.	Research process and findings discussed prompting thoughts to deepen insights.
Embedded in EMS organizational life	5 years	To allow for deeper understanding of everyday EMS reality,	Trust built through relationships developed as part of embedded researcher role enabled the research.

The interviews sought to capture the diverse experiences of the full range of EMS actors involved in the PP experience. Since the resilience capacities can be hard to examine from interviews alone, a series of observations to allow for further exploration, and to prompt further questions, were done. Observations and wider reflections were recorded in a research diary.

### Data analysis

**Figure 2.**
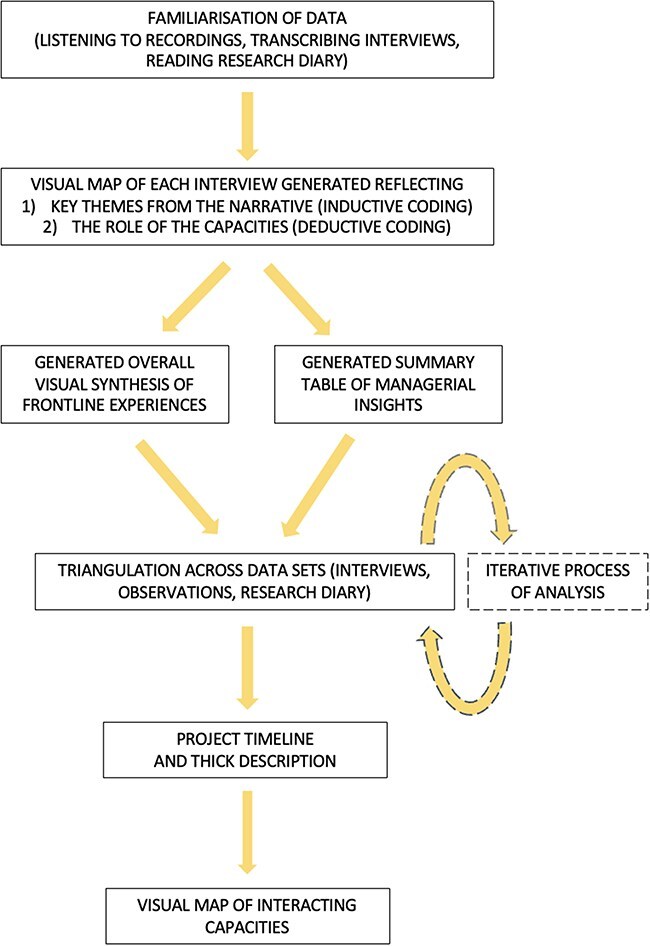
Summary of analytic steps

Guided by principles that recognize both ‘rigour and imagination’ (Green and Thorogood, 2004) as important, the analysis sought to work with the data visually and systematically by taking the steps outlined below (see Figure 2).

Following usual practice (Green and Thorogood, 2004), the first step was familiarization with the data (listening to interviews, transcription of interviews and reading research diary). Next, a visual map (see the Supplementary Material) of each interview was created drawing on a combination of deductive and inductive coding (Fereday and Muir-Cochrane, 2006). High-level inductive coding of interviews identified the main themes of individual narratives and deductive coding, using the conceptual framework as a guide, explored if and how the capacities were revealed as influencing the experience.

As the influence of positional power and hierarchy was evident from interview data, managerial and frontline perspectives were separated. Given commonalities of experience, a visual synthesis of all frontline interviews was created to reflect their overall experience. In addition, a summary table of key managerial insights was generated, highlighting themes across interviews, to better understand any differences between managers. There were no significant differences in the experiences reported in detail in this paper.

Next, triangulating across interview sets and observations, an overall timeline of the PP and a thick descriptive narrative of the experience was created. Discussions of this narrative within the wider research team were part of the validation process. Finally, analytic insights about the capacities, their interactions and their influences on the experience were summarized visually. Although presented in Figure 2 as a linear process, this analysis process involved cycles of iteration (Green and Thorogood, 2004).

Several principles to enhance research rigour were applied. These included triangulation across multiple datasets, regular research team debriefing and support, member checking with the PP crews, the use of theory and an audit trail of data collection and analysis (Gilson *et al*., 2011). As is good practice in ethnographic methods, a thick description (Yanow *et al*., 2009) of the findings was also initially developed as part of the case study report, and this was the basis for subsequent analysis. Lastly, while the period of study for the PP was only 6 months, the first author (L.B.) spent 3 years as an embedded EMS before data collection for this study began. This represents the prolonged engagement known to support depth of understanding (Gilson *et al*., 2011). At the same time, constant reflection on the ways in which L.B.’s position might influence the research was enabled through the use of a research diary (Robson, 2002; Green and Thorogood, 2004) and regular discussions with the broader research team (Fitzpatrick, 2019).

## Results

### The timeline of the PP

**Figure 3.**
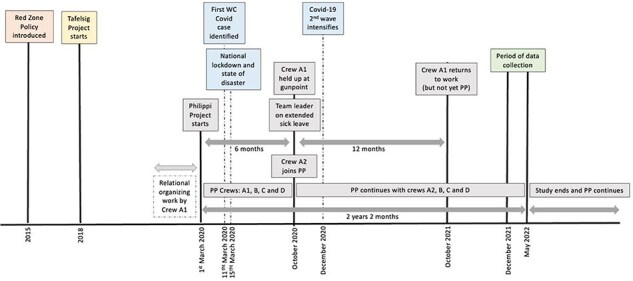
PP timeline of events

The PP took inspiration from an earlier, similar project implemented in Tafelsig, a neighbouring Cape Town community and also a red zone area (Figure 3). Learning from this experience, the frontline paramedic brought the idea of a dedicated community-based ambulance to Philippi, his own community (Crew A1, 15 February 2022).

Box 1: EMS day-to-day functioning

EMS operates on a shift system, with four shifts (A, B,C and D) in total, each of which is also divided into 12-h day and night shifts (e.g. A1 and A2). All ambulance crew staff and staff in the communications centre are rostered into these four shifts, rotating through the day and night shifts on a 4-week cycle. An ambulance crew comprises of two people who routinely work together. At the start of each shift, ambulance crews report for duty at the ambulance base and wait to be dispatched within their allocated district to their first call by the communications centre. Crews on different shifts generally only see each other at ‘parade’ when the shift leader holds a short meeting at the shift changeover (7 am or 7 pm) and there are no routine spaces that bring people across the different shifts (A–D) together. The ‘Liverpool formula’ is used within the Western Cape EMS to determine the crew mandate (PC 4 May 2022). It shows that running an ambulance 24/7 requires 10 people (or five crews) and takes into account both annual leave and illness over a 1-year period when considering appropriate staffing of the ambulance.

The EMS communication centre plays an integral role in ambulance operations. Also known as ‘the control room’, this centre receives all calls from members of the public who require an ambulance. The call-taker captures the patient’s details, triages them according to severity of their condition, and then sends the call to the relevant district dispatcher electronically. The dispatcher then sends an ambulance from that specific district giving priority to the most urgent calls.

From 2019, this PP team leader took on the initial relational organizing work (Figure 3) of building three key sets of relationships. First, among EMS colleagues living or working in Philippi who had quite specific characteristics and skill sets that had little to do with their clinical qualifications. Five crews (Box 1) who were ‘down to earth’ (Crew A1, 15 February 2022) with community links and community engagement skills were recruited. Second, relationships were built with the South African Police Services (SAPS). The idea was pitched in each police station by saying *Listen guys, we can help each other* (Crew A1, 15 February 2022), to try and get a sense of who would be willing to participate. Third, using existing personal community links as a starting point, efforts were made to foster support for the idea among community-based groups in Philippi.

**Figure 4.**
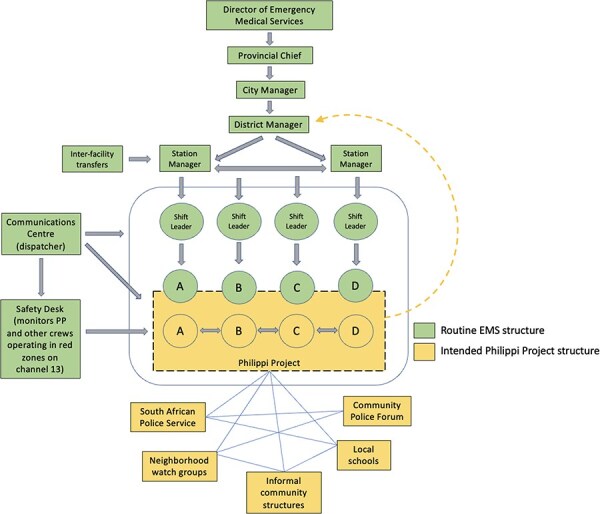
Intended PP organization within EMS hierarchy and desired community network

Once support from crews and SAPS had been secured, the district manager was approached to discuss the PP idea. With her approval, a dedicated ambulance was then allocated to the PP, and given new equipment (Crew A1, 15 February 2022; District manager, 14 March 2022). To allow some degree of self-organization, the PP crews were permitted to set their own hours (as long as a there was a crew available 24/7) and to report in Philippi (where many of them lived) rather than at the ambulance base, as is the norm (Crew A1, 15 February 2022, Crew C, 23 March 2022, District manager, 14 March 2022). In addition, engagement with the ‘control room’ (see Box 1) ensured that all calls made from Philippi for EMS support were dispatched to the PP team, instead of sending the next available crew irrespective of the geographic location (Crew A1, 15 February 2022; District manager, 14 March 2022). The PP was also linked to the Safety Desk staff who offer support to crews while in red zones. The intention was that the PP team would function with some degree of autonomy, even while located within the existing EMS hierarchy. So, as Figure 4 demonstrates, the PP team leader engaged directly with the district manager (dotted arrow) for support and assistance, instead of following the usual chain of command of instructions and reporting through the shift leader to station manager and then the district manager. It was also intended that the PP would be networked with other Philippi-based services and groups (Figure 4), with the idea that multiple mutually beneficial relationships with community-based groups would help to build trusting relationships and improve crew safety.

The PP was formally launched on 1 March 2020, 2 weeks before the first case of COVID-19 was identified in the Western Cape (Figure 3). A strong sense of passion and purpose was quickly re-ignited within the PP crews when the project started (Crew A1, 15 February 2022; District manager, 14 March 2022) and this sustained them through the first 6 months of the COVID pandemic (Crew A1, 15 February 2022; District manager, 14 March 2022; SL, 17 April 2022). While the pandemic had a significant impact on the health system as a whole, ambulance crews on the frontline were at particular risk given the nature of their work. Yet the PP crews continued working at a *time [where] nobody wanted to be on an ambulance* (District manager, 14 March 2022), and when ambulance crews at other bases had downed tools due to personal protective equipment shortages and unsafe working conditions.

In October 2020, however, Crew A1 were held up at gunpoint in Philippi (Figure 3). The crew suffered significant trauma and could only return to work 1 year later (Crew A1, 15 February 2022; District manager, 14 March 2022). The assault had ripple effects across the PP crews, and EMS at large. *We were all a bit bruised after that* (District manager, 14 March 2022). A new crew was appointed, and the PP continued *but it was not alive in the same way* (Crew A1, 15 February 2022). EMS managers noted that *when [the PP team leader] stepped back, they weren’t as united* (SL, 17 April 2022) and it *felt like there was a missing link* (District manager, 14 March 2022).

Over the next 18 months, the project continued but some of the initial gains in the PP were difficult to sustain. The crews focused on strengthening relationships with SAPS, to secure police escorts more easily (Crew A1, 15 February 2022; Crew C, 23 March 2022). With no officially appointed team leader, the project’s relative autonomy within EMS was no longer protected. Over time, the PP reverted to the usual EMS routines, and it became little more than a dedicated crew for the Philippi area (Crew D, 15 February 2022; District manager, 14 March 2022). The initial goal of a community-based ambulance service was lost.

### Understanding how the PP evolved over time

#### The early months—critical capacities enabling collective action

**Figure 5.**
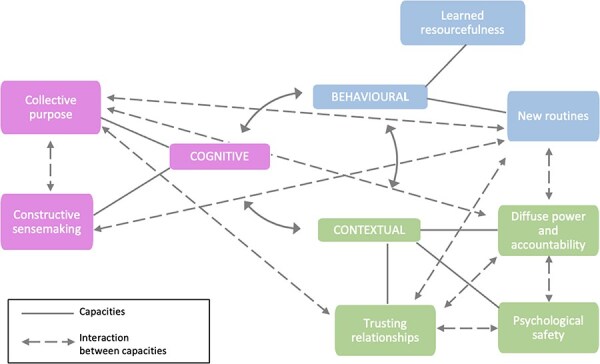
Capacities enabling collective action during initial stages of the PP

The capacity attributes that enabled the launch and initial work of the PP are summarized in Figure 5.

Even prior to its official launch, as described, Crew A1 laid the ground for the PP through the largely invisible, but intentional, development of relationships, also demonstrating learned resourcefulness in drawing from the Tafelsig experience. In the early months of the project, the PP team leader worked to build excitement, and a sense of collective purpose among the crews, focused on improving the services offered to the community. The district manager observed that at the introductory meeting: *we met early morning one day in the parking lot, and there was such excitement for this thing to start…we all saw the same thing* (District manager, 14 March 2022).

The sense of collective purpose was a critical catalysing factor for the PP but was moulded from slightly different starting points. The PP team leader was specifically motivated by the need to address the inequity faced by the residents of Philippi, and to work with relative freedom and autonomy from the organizational EMS constraints to better able to meet community needs (Crew A1, 15 February 2022). Reflecting on the Tafelsig experience, he noted that the existing levels of care were unacceptable *we cannot hide behind the fact that this area is a red zone… because of the robberies people weren’t getting the services they should get from government. There was a need to do something* (Crew A1, 15 February 2022). Other crew members were more strongly motivated by the need to improve the relationship with SAPS (Crew A2, 18 February 2022). They judged that having a dedicated Philippi crew would enable improved connections with SAPS, making it easier to get police escorts, which would in turn improve response times. For other crews, having a dedicated ambulance for Philippi could lead to improved relationships with the Philippi community and thereby reduce the attacks on ambulance crews (Crew A2, 18 February 2022; Crew C, 23 March 2022). Once engaged, the district manager, meanwhile, also felt that an ambulance *that belonged to the community* would enable crews to do more creative problem solving at the front line (District manager, 14 March 2022).

Given the fragmentation of the shift system (Box 1), building collective purpose also required deepening trusting relationships among crews across shifts through some new routines (Box 1). The PP team leader purposefully moderated a very active WhatsApp group that functioned as a space both for nurturing relationships and keeping everyone informed [PP team leader] *was good about building relationships between the shifts* (Crew D, 15 February 2022). Furthermore, this communication channel, and regular visits with other crews, created spaces for constructive sense-making about project intentions and challenges.

As noted earlier, deliberate efforts were taken to adapt usual EMS routines to support the PP and to allow some degree of self-organization—such as in the way the control room allocated new calls to crews. The arrangements and their negotiation demonstrated both diffused power and accountability and some sense of psychological safety, both of which also enable trusting relationships. Feeling empowered, Crew A1 worked outside of usual hierarchies to initiate the PP, bypassing the shift leader and station manager, and working closely with the District Manager in developing new practices to maintain the PP’s relative autonomy, bringing any *challenges straight to the boss Mama at the back* (SL, 17 April 2022). The PP leader had the type of personality that was required for this: *he was very vocal, he was straightforward, he was unapologetic* (SL, 17 April 2022). The District Manager also felt sufficient psychological safety in the organization to support the PP—e.g. exercising her own power by enabling meetings between senior EMS managers and SAPS station commanders to formalize the relationship between EMS and SAPS (District manager, 14 March 2022). Efforts taken within EMS since 2015 to flatten hierarchies and develop distributed forms of leadership with delegated decision-making power, creating spaces for frontline staff to suggest creative ideas to strengthen EMS, supported the PP’s emergence (District manager, 14 March 2022).

Finally, the first few months of the project benefitted from the way the PP leader was embedded in key Philippi community groups, with trusting relationships. He cited many informal examples of their support. In one instance, during an attempted hijacking, a community member offered their home as a safe house for the ambulance crew (Crew A1, 15 February 2022).

#### The gravitational pull back to EMS practices and routines that undermined the PP’s emergent ways of working

**Figure 6.**
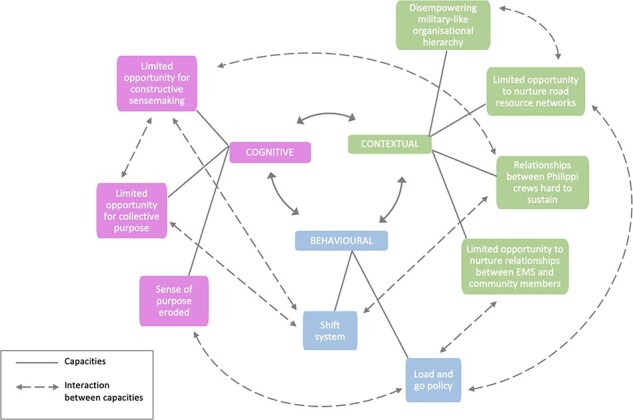
Capacity gaps restraining collective action over time

Over time, hierarchical practices and the usual EMS routines reasserted their influence, undermining the still emergent ways of working within the PP. These experiences highlight systemic capacity weaknesses that undermined collective action, as summarized in Figure 6.

Box 2: What is the ‘load and go’ policy’?

In order to improve the safety of ambulance crews, a ‘load and go’ policy was introduced in red zone neighbourhoods. Previously, crews could offer some clinical care in people’s homes, or at the scene of the accident in the ambulance itself. However, with an increasing number of assaults on ambulance crews, the guidance has become to simply ‘load’ the patient and ‘go’ to the hospital as quickly as possible. The ambulance crew needs to provide any medical treatment en route, although there are practical challenges with delivering care in a moving vehicle. Another option is to go to the nearest police station if the patient needs to be stabilized before they are transported to hospital, but in practice this does not happen often.

Despite prior attempts by senior managers to flatten hierarchies, the existing chain of command remained very much in place. Most PP crew members experienced the dominant organizational culture as one of following orders from senior managers: *we don’t make decisions on our own. We wait for the decision, and then we do* (Crew A2, 18 February 2022). Thus, when the PP leader stepped down following Crew A’s assault, the other crews largely returned to the former, more passive ways of working. *We like to follow what the bosses are saying, we don’t do as we please* (Crew A2, 18 February 2022). As the autonomy of the PP reduced over time, the diffuse power and accountability it had benefitted from initially was also lost. Instead, PP crew members increasingly felt bound to a largely disempowering organizational hierarchy.

Two key EMS routines that further undermined the PP were the shift system (Box 1) and the load and go policy (Box 2). Relationships across crews were particularly hard to sustain in the face of the shift system after Crew A1 left the project, and the initial WhatsApp group died: *now we don’t know what is happening in other shifts* (Crew D, 15 February 2022). By the time of these interviews, the PP crews had not been in the same space for 18 months. When asked how often they got to see each other, one crew member said: *When the boss mama introduced us, that was the first and last meeting* (Crew A2, 18 February 2022). As crews felt increasingly disconnected from each other, it became impossible to sustain opportunities for constructive sense-making and for nurturing the collective purpose that initially catalysed the PP. The ‘load and go’ policy also had negative impacts on these cognitive capacities needed to support collective action (see Figure 6). Although intended to keep ambulance crews and SAPS safe, it meant that the PP crews had very little time on scene and were not able to use their clinical skills to stabilize or treat patients. As ‘saving lives’ is a defining feature of the profession, this routine eroded the crews’ sense of purpose in relation to the PP and, consequently, their capacity for collective action. At the same time, the initial relative autonomy of the PP itself had generated challenges undermining collective purpose. For example, because they were no longer present at some of the EMS shared meetings (such as the morning parade where all crews come together when reporting for duty), PP crews began to feel excluded from some of the opportunities discussed there, like openings for further study (Crew A1, 15 February 2022). The failure to reward PP crews appropriately may have contributed to their declining enthusiasm for the initial PP model (Crew A1, 15 February 2022).

The ‘load and go’ policy, moreover, also limited opportunities to nurture broad resource networks while on duty, in terms of relationships between the EMS and the community members. In one instance, a neighbourhood watch group offered to escort a PP crew to the patients’ home, but as they were already being escorted by SAPS, they could not stop and chat, or exchange phone numbers for future assistance (Crew C, 23 March 2022). Similarly, attempts to extend or connect into community networks (e.g. visiting a local community kitchen) were halted because, according to red zone protocol, a PP crew *needs a SAPS escort* (Crew C, 23 March 2022). Over time, the crews increasingly prioritized building relationships with SAPS over those with community groups. Although managers reported that they would celebrate crews who built community relationships, many crews simply did not feel sufficient psychological safety to take such initiative in the face of what they experienced as the disempowering, military-like nature of EMS. Instead, this pre-existing organizational culture primed them to follow orders.

#### Why were relationships with SAPS strengthened over community relationships?

Although crews had various starting points for getting involved in the PP, the need to improve relationships with SAPS to secure police escorts was important for some and only grew stronger after the 2020 assault (Crew A1, 15 February 2022; Crew C, 23 March 2022). This collective purpose came to drive practice, rather than collective action with community members. Initially, the DM *put the message out that it was important for them to find a place in the community for our ambulance…and make the community understand that it’s theirs… But, after [the PP team leader] got assaulted I never re-visited the concept* (District manager, 14 March 2022).

Recognizing that SAPS had competing priorities—*I don’t blame them, they have their own work to do* (Crew C, 23 March 2022)—a common strategy was for EMS crews to assist with SAPS priorities first, and then get assistance with EMS work: *first we help them out, and then they help us out* (Crew A1, 15 February 2022). In practice, this meant telling the control room to hold patient calls, with unknown consequences for patients, while they assisted SAPS in signing off death declarations (Crew A2, 18 February 2022; C, 23 March 2022; D, 15 February 2022). Over time, the relationships between PP crews and local SAPS stations improved (Crew A2, 18 February 2022; Crew D, 15 February 2022). *The way they were treating us before… it was bad. At least now they are trying* (Crew C, 23 March 2022). Some crews reported their friendships with SAPS as the reason for reduced times waiting for SAPS escorts (Crew A2, 18 February 2022; Crew C, 23 March 2022; Crew D, 15 February 2022). The control room also noticed a similar trend *some crews have developed good relationships with SAPS…I can see it because when A [Crew A2] is at Phillip East SAPS, they don’t wait long at all* (Safety desk, 18 April 2022).

However, as one crew reflected: *We have good relationships with SAPS, but not with community groups yet* (Crew A2, 18 February 2022). Three other factors explain this situation and demonstrate the complexities of sustaining EMS–community connections.

First, although most of the PP crews were recruited because they lived or had lived or worked in Philippi, their personal experience simply did not bring strategic connections and organizational benefits. When the PP team leader left the project, there was little intentional relationship building work at the community level. Those crews involved in community activities often did so in their personal capacity, e.g. volunteering at a local community gym (Crew D, 15 February 2022). Others did not want to be known as working for EMS. One PP crew member specifically opted to work outside his own neighbourhood for safety reasons and even removed his green uniform before returning home to try and keep his EMS identity anonymous *I am staying in Khayelitsha*^1^*, but I don’t want to work that side When I get off duty I take off my [uniform] for safety reasons. I don’t want to work that side, I can’t work in my community where I grew up. I would like to, but no, not with this job that I am doing. I can’t* (Crew A2, 18 February 2022).

Second, the COVID-19 pandemic meant *the ability to connect with community groups was hampered. There were lots of plans that people shared, but then they couldn’t take them forward because of regulations that you couldn’t have community meetings* (District manager, 14 March 2022). Many community activities and meetings were cancelled due to restrictions on gatherings introduced as part of the National State of Disaster regulations (Goverment N, 2020). Stigma during the pandemic also undermined community connections, given the widespread belief that ambulances were spreading the virus. PP crews reported patients expressing the fear that ambulances had *come to kill our people* (Crew A2, 18 February 2022). There were multiple reports of crews being turned away from homes, or patients running from the ambulance for fear of being taken away to die (Whyle *et al*., 2020). Many community groups were just not keen to work with EMS during this time. There was a general sense that some of these connections were stronger before COVID-19, but had been lost: *we need to go and ask for a seat at the table…and make ourselves visible again* (District manager, 14 March 2022).

Third, as noted, internal EMS organization routines and practices themselves created obstacles to building community connections. The ‘load and go’ policy limited opportunities for engagement, while, reflecting passivity in the face of hierarchy, community engagement activities were commonly seen among EMS crews as *the job of the manager* (SL, 17 April 2022). Where crews did take the initiative to form connections, they did so with caution as they felt they *have to get permission for any community engagement…and don’t want to be in trouble. Let’s do our core function, treat patients, go to SAPS* (Crew A2, 18 February 2022). Community engagement activities were, then, largely seen as additional to their primary role, and outside their scope of practice.

## Discussion

The PP embodies the spirit of earlier international EMS initiatives such as the 1967 Freedom House Ambulance service in the USA, which sought to address issues of inequity in access to emergency care due to racial segregation (Edwards, 2019; 2021). This service led to the first paramedic textbook ‘Emergency care in the Streets’ (Caroline, 1979), inspiring the US national ambulance service. The Freedom House experience illustrates the transformative potential of community-oriented models of care such as the PP—which was developed by frontline ambulance crews as a collective response to the complex health system challenges of inequality and violence in Cape Town. It was hoped that, in time, the new forms of collaboration entailed by the PP might support new ways of working across the wider EMS. However, despite the early energy and achievements, challenges emerged, which made it hard to sustain the PP as originally envisaged. Although it continued to exist beyond the period reported in this paper, it lost its initial goal of becoming a community-based ambulance and became little more than a dedicated ambulance for the Philippi area. Nonetheless, as an unusual empirical example of a community-oriented model of EMS care, this paper offers globally relevant insights.

The case demonstrates, first, that ‘chronic stress is an enduring feature of the lived reality of health systems’ (Gilson and Barasa, 2024, p. 44) and the acute shocks of disease outbreaks, climate catastrophes or economic crises inevitably overlay such stress (Barasa *et al*., 2017; Kagwanja *et al*., 2020; Gilson and Barasa, 2024). As demonstrated here, while the PP emerged as a potentially transformative response to the everyday stressors of inequality and societal violence, it was also impacted at an early stage of its development by two acute shocks—namely, the COVID-19 pandemic and the hijacking of the team leader (a specific incident of societal violence)—that ultimately restricted and undermined its implementation.

Second, the application of the EHSR lens in analysing this experience helps to reveal the capacity attributes influencing the PP’s implementation and offers insights about how to initiate and sustain community-based EMS models of care. As also seen in other resilience studies (Gilson *et al*., 2017; Kagwanja *et al*., 2020; Waithaka *et al*., 2020), the analysis shows that while individuals can initiate responses to stress and shock, longer-term strategies require collective engagement and action to be sustained, suggesting that this is key to transformative resilience.

The PP experience reveals that in developing new ways of working, EMS frontliners were supported by forms of higher-level leadership that provided a delicate balance of freedom to experiment, and ongoing support and mentorship. At the same time, distributed forms of leadership, which share decision-making power among multiple actors across the system rather than restricting such power to those in managerial positions (Gilson *et al*., 2024), were critical. Enabling organizational routines was also important in underpinning collective action, and interacted with each other. For example, in the earlier period, new routines (behavioural capacity) introduced by the PP team leader created the space for constructive sense-making (cognitive capacity) in an otherwise fragmented system, and this nurtured collective purpose (cognitive capacity) and trusting relationships (contextual capacity) within the PP team. This reinforcing loop among capacity attributes was key to the initial success of the PP. Wider EHSR literature affirms that leadership practices (Gilson *et al*., 2020) can create the space for actors to take transformative actions that can nurture systemic capacities, and that transformative responses to stress are supported by the shared meanings (sense-making) that drive new ways of acting collectively (Kagwanja *et al*., 2020).

Over time, however, a combination of societal shocks (COVID-19 and ongoing societal violence) and pre-existing factors in the organizational context resulted in a gravitational pull back to usual EMS practice. More specifically, frontline staff were no longer comfortable taking risks and countering the dominant norm of ‘following orders’ and challenging the culture of hierarchy (Gilson *et al*., 2014; Nxumalo *et al*., 2018). In addition, the limited pre-existing links and networks between EMS and community groups posed particular challenges for the sustainability of this community-based EMS model of care.

The PP experience suggests, therefore, that organizational routines in the Western Cape EMS are still perhaps best suited to supporting the existing hospi-centric model of care (that prioritizes transporting patients to hospitals) rather than community models of care such as the PP. Yet, it also illuminates the types of systemic capacities needed to nurture new transformative models of care and support the emergence of resilience over time (Gilson and Barasa, 2024). As reflected in wider literature, it is important to create organizational opportunities for relationship building, constructive sense-making, psychological safety and diffusing power and accountability (Lengnick-Hall *et al*., 2011). These, in turn, create spaces for developing collective purpose, and nurturing new ways of working (Saulnier *et al*., 2022). Working with communities also specifically demands community engagement skills. Health actors must take the time needed to nurture such relationships, recognizing community members as partners, not beneficiaries (Barker *et al*., 2020). These interacting sets of resilience capacities are embedded in people, teams and system routines (Gilson *et al*., 2024) and must be spread across system levels (Lengnick-Hall *et al*., 2011; Wiig *et al*., 2020; Gilson and Barasa, 2024).

### Study limitations

Our research aimed to understand the PP experience drawing on a range of perspectives within EMS. However, SAPS members were not invited to participate, and attempts to identify community members as part of the PP were unsuccessful. These missing views would have shed light on the experience and could be the focus of future research in this area.

L.B.’s positionality may have meant that an element of social desirability bias may have influenced interview responses. While the option of refusing to be interviewed was offered, it would have been highly unlikely for anyone to take up this option, given the clinical and bureaucratic hierarchies. However, the research participants did not appear to be constrained in their responses and instead seemed to appreciate the research interview as a debriefing space. In some instances, the participants felt comfortable enough to share their fears of intimidation—one crew requested an off-site interview. Constant reflection on the ways in which L.B.’s position might influence the research was considered throughout the research process, using a field note diary and regular discussions with the broader research team.

## Conclusion

Changing contextual features, such as the shifting burden of disease and wider societal challenges, impose stress and shocks on EMS in South Africa and globally. The existing routines, which were established to support a hospi-centric service, are no longer sufficient, and instead, a community-based model of care represents a potentially transformative strategy that can enhance EMS resilience to the stressors experienced when working at the community–health system interface. However, in an organization infused with clinical and other hierarchies, it is important to create and protect space for frontline staff to develop creative, collective solutions to such stress. Time and permission must also be granted to nurture community-level relationships and specific strategies that protect the space for frontliners to develop creative solutions to the daily stressors of violence and inequality are essential.

## Supplementary Material

czae070_Supp

## Data Availability

The data underlying this article are available in the article and its online supplementary material. The interview data underlying this article cannot be shared publicly to maintain the privacy of the individuals who participated in the study.

## Footnotes

1. Khayelitsha is 22 km from Philippi and has similar red zone status. Like in Philippi, many Black families were relocated to Khayelitsha during the Group Areas Act. Today, the neighbourhood has a mix of formal and informal housing, but poverty is widespread.

## Reflexivity statement

This research was done by a team of practitioners and researchers who have been embedded in the health care system of the Western Cape Province for over a decade. All authors are committed to knowledge co-production to support health systems development. This paper reflects one output of a broader body of work that seeks to bridge the research to policy and practice gap. All authors live and work in Cape Town, South Africa. The collective work reflects the diverse positionalities of the authors who include two practitioners at differing levels of organizational hierarchy (S.H. and S.D.V.). L.B., the lead author, completed this work as an embedded researcher nested in EMS in order to gain a deeper understanding of the organizational dynamics and priorities. She worked closely with frontline ambulance crews and validated research findings with them to adequately reflect their experiences. She received support from two senior researchers L.G. and A.G. Four of the five authors are female. Three of the five authors are people of colour.
