# Supplementary figures and images for: A community-based ambulance model: lessons for emergency medical services and everyday health systems resilience from South Africa
Source: Health Policy Plan. 2024 Aug 2;39(9):956–69. doi: 10.1093/heapol/czae070 (PMC11969215; doi:10.1093/heapol/czae070)

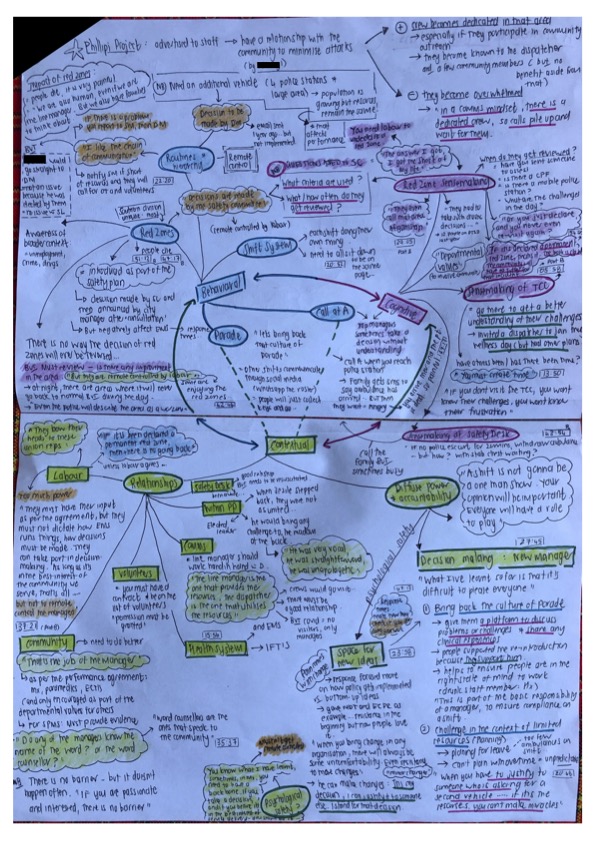

Supplement: czae070_Supp [file czae070_supp.zip › Visual map example_anonymised.jpg]
